# The genome sequence of the hawthorn shieldbug, *Acanthosoma haemorrhoidale* (Linnaeus, 1758)

**DOI:** 10.12688/wellcomeopenres.17926.1

**Published:** 2022-07-05

**Authors:** Liam M. Crowley, University of Oxford and Wytham Woods Genome Acquisition Lab, Darwin Tree of Life Barcoding collective, Wellcome Sanger Institute Tree of Life programme, Wellcome Sanger Institute Scientific Operations: DNA Pipelines collective, Tree of Life Core Informatics collective, John Mulley, Darwin Tree of Life Consortium

**Affiliations:** 1 Department of Zoology, University of Oxford, Oxford, Oxfordshire, UK; 2 School of Natural Sciences, Bangor University, Bangor, LL57 2UW, UK

**Keywords:** Acanthosoma haemorrhoidale, hawthorn shieldbug, genome sequence, chromosomal, Arthropoda

## Abstract

We present a genome assembly from an individual male *Acanthosoma haemorrhoidale* (hawthorn shieldbug; Arthropoda; Insecta; Hemiptera; Acanthosomatidae). The genome sequence is 866 megabases in span. The majority of the assembly (99.98%) is scaffolded into 7 chromosomal pseudomolecules with the X and Y sex chromosomes assembled. The complete mitochondrial genome was also assembled and is 18.9 kilobases in length.

## Species taxonomy

Eukaryota; Metazoa; Ecdysozoa; Arthropoda; Hexapoda; Insecta; Pterygota; Neoptera; Paraneoptera; Hemiptera; Heteroptera; Panheteroptera; Pentatomomorpha; Pentatomoidea; Acanthosomatidae; Acanthosoma; Acanthosoma haemorrhoidale (Linnaeus, 1758) (NCBI:txid483950).

## Background

The hawthorn shield bug, *Acanthosoma haemorrhoidale*, is a large Pentamoid shield bug, easily recognisable by their size (typically 13mm or more in length) and bright green and red coloration. The species is common on hawthorn ( *Crataegus monogyna*), where the berries comprise their principal food source, but are also found in mixed woodland and will feed on leaves of oak, hazel, and other deciduous trees and shrubs. Adults overwinter in leaf litter or under bark, and sometimes in buildings, and emerge in spring. Eggs are laid in several batches in late spring to early summer, and females exhibit no maternal care, unlike other members of the *Acanthosomatidae* ( Hanelová & Vilímová 2013; Tsai & Rédei, 2015). First ecdysis to adult emergence takes around 35 days ( Hori *et al.,* 1993).

Originally classified as *Cimex haemorrhoidalis* by Linnaeus in 1758, the genus Acanthosoma (acantho- = spiny, -soma = body) was raised by Curtis in 1824 for the spined keel on the ventral surface ( Curtis, 1824). The species name references the blood red coloration and appearance of discharging blood, particularly from the tip of the abdomen. The species has a trans-palaearctic distribution and comprises at least three currently-recognised subspecies: *A. h. haemorrhoidale*, Linnaeus 1758; *A. h. angulatum*, Jakovlev 1880; *A. h. ouchii*, Ishihara 1950 ( Tsai & Rédei, 2015).

The classic work by Southwood and Leston on British land and water bugs ( Southwood & Leston, 1959) describes a distribution across much of England and Wales, with only recent colonisation of Northern England. Whilst sporadic records for Scotland are found from the mid-20th century, it appears that a gradual northward range expansion has been underway from at least the mid-1990’s, and that the species is now well-established across northern England and is reasonably common up to the central belt of Scotland, with more scattered reports from further north ( Ramsay, 2014). A similar northern expansion appears to have occurred in Finland in the mid-20th century ( Ramsay, 2014; Southwood & Leston, 1959), and it may be interesting to investigate parallel behavioural or physiological changes in these northward-bound populations. Development is temperature sensitive, with high mortality at 30°C ( Hori *et al.,* 1993), and more southern parts of the species range may therefore become unsuitable in the future.

In contrast to groups like the Lepidoptera, where females produce pheromones to attract males, in the Pentatomoidea it seems to be males that produce pheromones, most likely to avoid parasitoids utilising female pheromones to find hosts, and male *A. haemorrhoidale* possess extensive abdominal sternal glands ( Staddon, 1990). The genome sequence will facilitate identification of biosynthetic pathways underlying pheromone production and reception in this species. Similarly, genomic data will shed light on host-symbiont relationships, including not only characterisation of bacterial symbionts themselves, but also the anatomical and behavioural mechanisms for storing and transmitting them to the next generation, such as the midgut crypts and the lubricating organs of females ( Kikuchi *et al.*, 2009).

Southwood and Leston report the diploid (2N) karyotype of *A. haemorrhoidale* to be 12, comprising ten autosomes and two sex chromosomes (XX or XY), and this accords with reported modal numbers for other members of the Acanthosomatidae ( Kaur & Patial, 2017; Rebagliati *et al.*, 2005; Southwood & Leston, 1959).

## Genome sequence report

**Figure 1.**
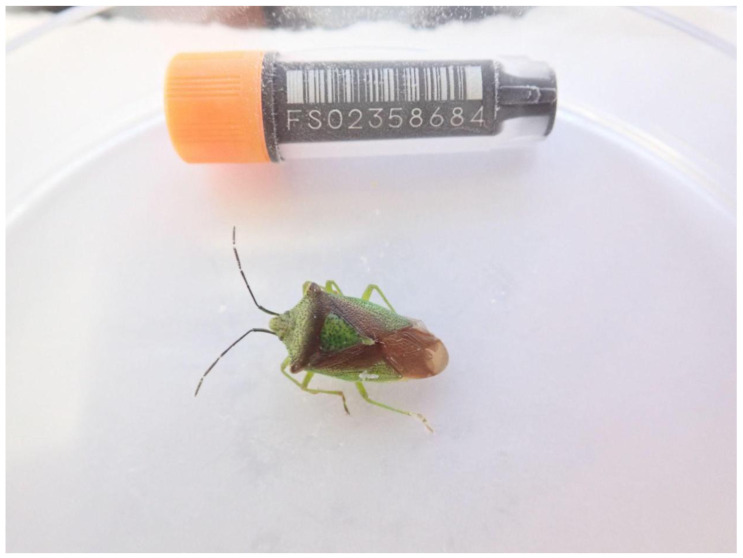
Image of the *Acanthosoma haemorrhoidale* specimen taken prior to preservation and processing.

The genome was sequenced from a single male *A. haemorrhoidale* collected from Wytham woods, Berkshire, UK ( Figure 1). A total of 26-fold coverage in Pacific Biosciences single-molecule HiFi long reads and 223-fold coverage in 10X Genomics read clouds were generated. Primary assembly contigs were scaffolded with chromosome conformation Hi-C data. Manual assembly curation corrected 321 missing/misjoins and removed 4 haplotypic duplications, reducing the assembly size by 0.08% and the scaffold number by 65.5%, and increasing the scaffold N50 by 112.02%.

**Figure 2.**
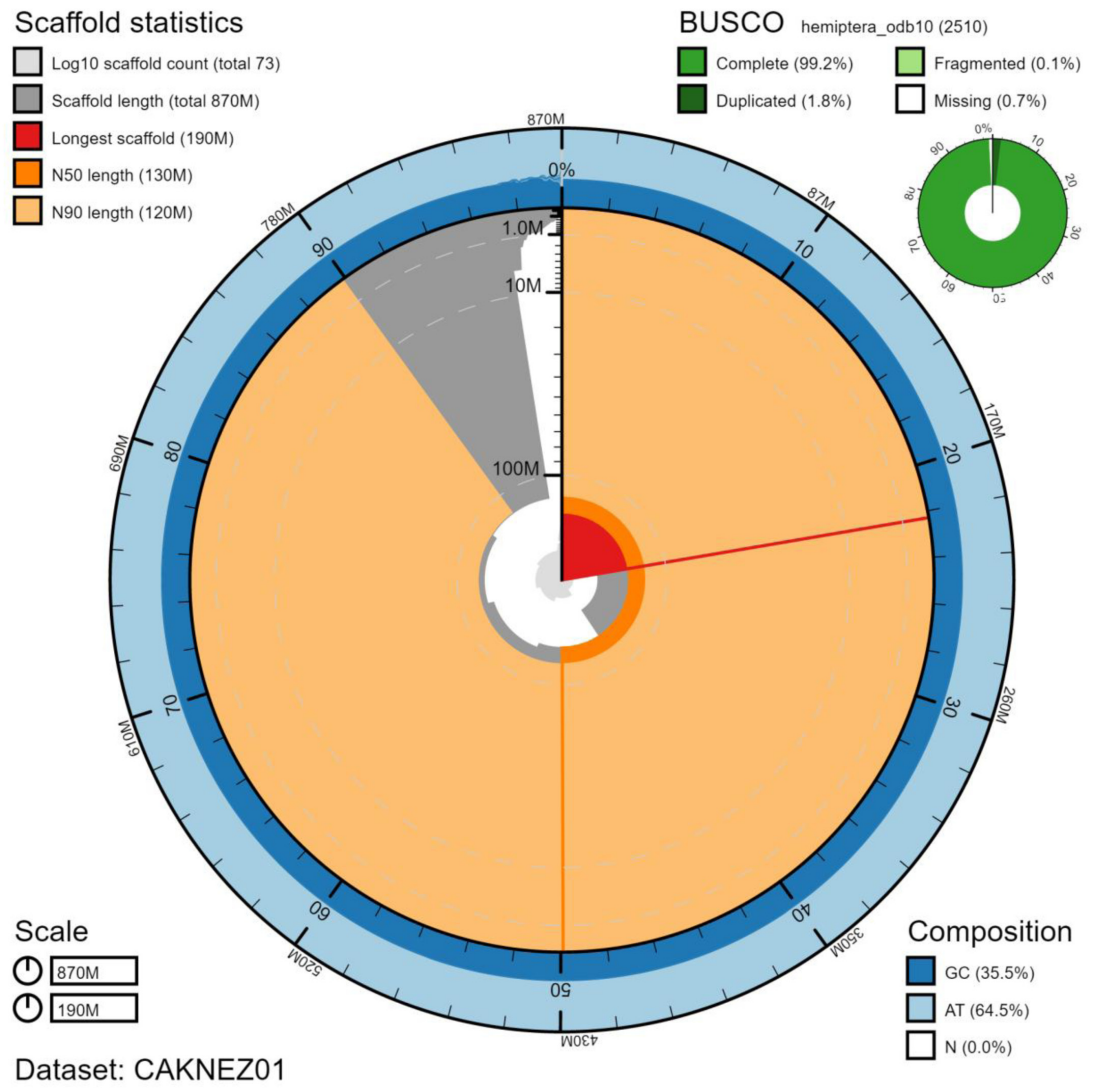
Genome assembly of *Acanthosoma haemorrhoidale*, ihAcaHaem1.1: metrics. The BlobToolKit Snailplot shows N50 metrics and BUSCO gene completeness. The main plot is divided into 1,000 size-ordered bins around the circumference with each bin representing 0.1% of the 865,622,309 bp assembly. The distribution of chromosome lengths is shown in dark grey with the plot radius scaled to the longest chromosome present in the assembly (193,544,673 bp, shown in red). Orange and pale-orange arcs show the N50 and N90 chromosome lengths (129,246,741 and 116,641,292 bp), respectively. The pale grey spiral shows the cumulative chromosome count on a log scale with white scale lines showing successive orders of magnitude. The blue and pale-blue area around the outside of the plot shows the distribution of GC, AT and N percentages in the same bins as the inner plot. A summary of complete, fragmented, duplicated and missing BUSCO genes in the hemiptera_odb10 set is shown in the top right. An interactive version of this figure is available at https://blobtoolkit.genomehubs.org/view/ihAcaHaem1.1/dataset/CAKNEZ01/snail#Filters.

The final assembly has a total length of 866 Mb in 72 sequence scaffolds with a scaffold N50 of 33.6 Mb ( Table 1). The majority, 99.98%, of the assembly sequence was assigned to 7 chromosomal-level scaffolds, representing 5 autosomes (numbered by sequence length) and the X and Y sex chromosomes ( Figure 2– Figure 5; Table 2).

**Table 1.** Genome data for *Acanthosoma haemorrhoidale*, ihAcaHaem1.1.

*Project accession data*	
Assembly identifier	ihAcaHaem1.1
Species	*Acanthosoma haemorrhoidale*
Specimen	ihAcaHaem1 (genome assembly; Hi-C)
NCBI taxonomy ID	483950
BioProject	PRJEB47321
BioSample ID	SAMEA8563710
Isolate information	Male, abdomen (genome sequencing); head/thorax (Hi-C)
*Raw data accessions*	
PacificBiosciences SEQUEL II	ERR6808043-ERR6808045
10X Genomics Illumina	ERR6688746-ERR6688753
Hi-C Illumina	ERR6688405
*Genome assembly*	
Assembly accession	GCA_930367205.1
*Accession of alternate haplotype*	GCA_930374635.1
Span (Mb)	866
Number of contigs	545
Contig N50 length (Mb)	3.36
Number of scaffolds	72
Scaffold N50 length (Mb)	129.2
Longest scaffold (Mb)	193.5
BUSCO * genome score	C:99.2%[S:97.4%,D:1.8%],F:0.1%,M:0.7%,n:2510

*BUSCO scores based on the hemiptera_odb10 BUSCO set using v5.1.2. C= complete [S= single copy, D=duplicated], F=fragmented, M=missing, n=number of orthologues in comparison. A full set of BUSCO scores is available at https://blobtoolkit.genomehubs.org/view/ihAcaHaem1.1/dataset/CAKNEZ01/busco#Filters.

**Figure 3.**
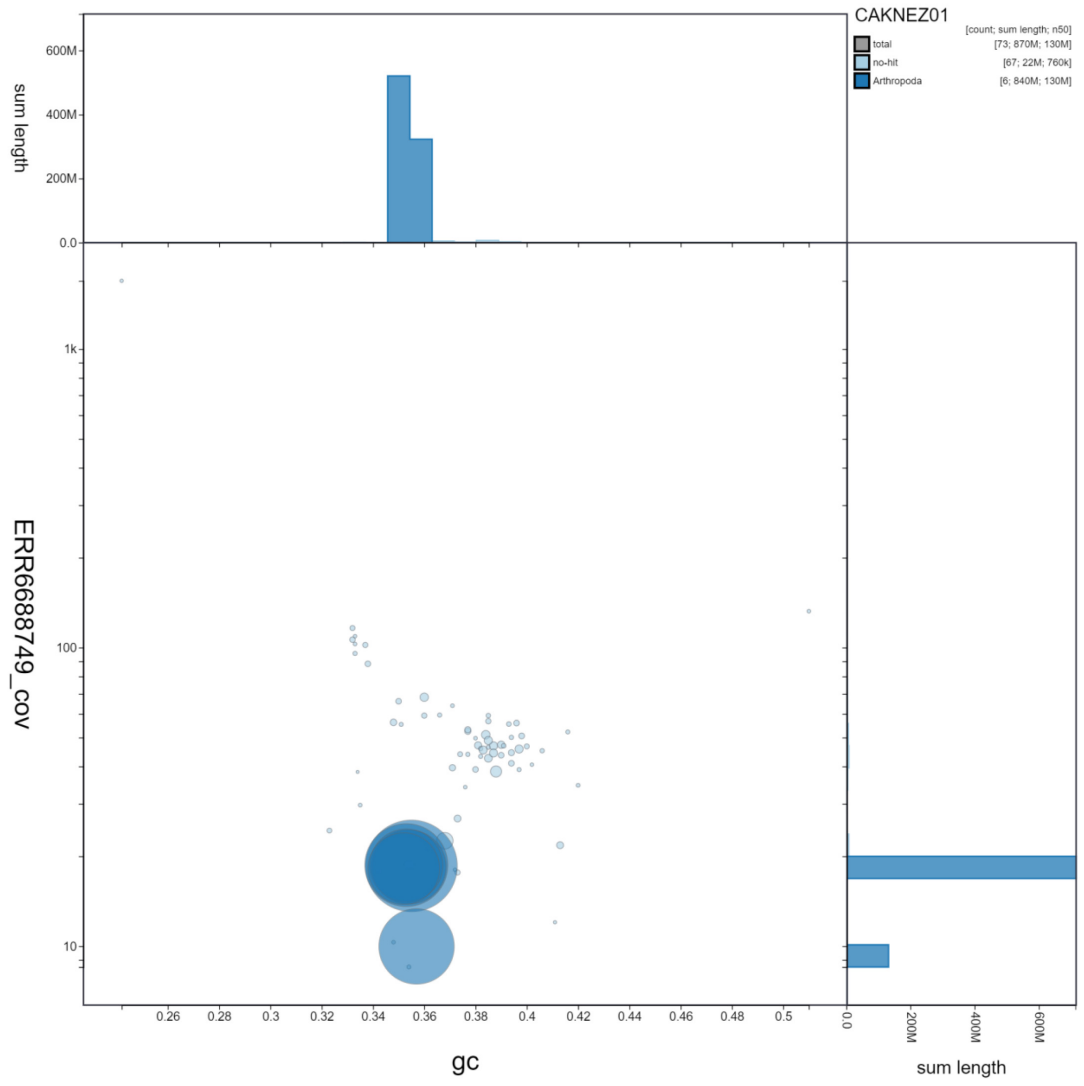
Genome assembly of *Acanthosoma haemorrhoidale*, ihAcaHaem1.1: GC coverage. BlobToolKit GC-coverage plot. Scaffolds are coloured by phylum. Circles are sized in proportion to scaffold length. Histograms show the distribution of scaffold length sum along each axis. An interactive version of this figure is available at https://blobtoolkit.genomehubs.org/view/ihAcaHaem1.1/dataset/CAKNEZ01/blob#Filters.

**Figure 4.**
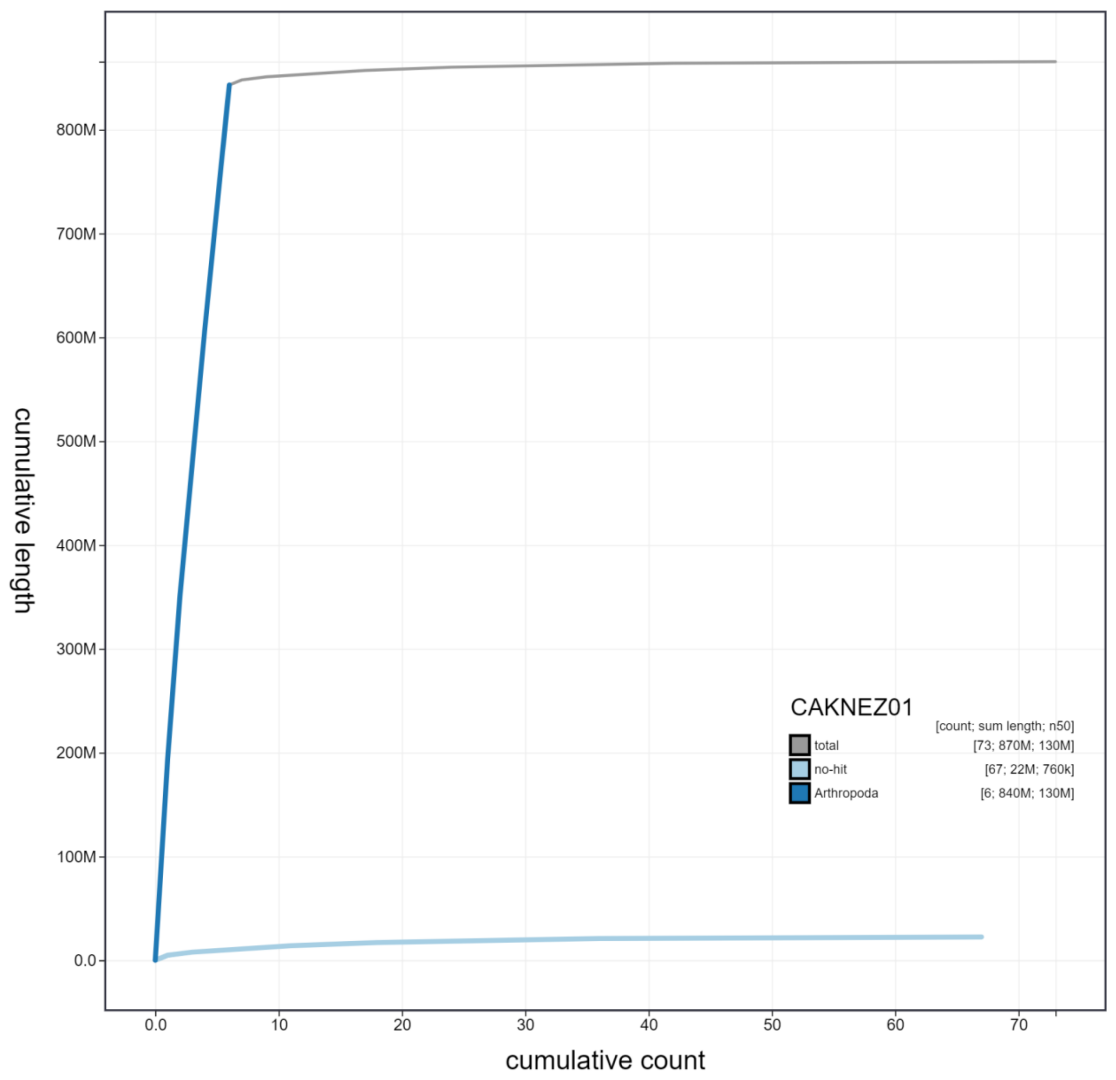
Genome assembly of *Acanthosoma haemorrhoidale*, ihAcaHaem1.1: cumulative sequence. BlobToolKit cumulative sequence plot. The grey line shows cumulative length for all scaffolds. Coloured lines show cumulative lengths of scaffolds assigned to each phylum using the buscogenes taxrule. An interactive version of this figure is available at https://blobtoolkit.genomehubs.org/view/ihAcaHaem1.1/dataset/CAKNEZ01/cumulative#Filters.

**Figure 5.**
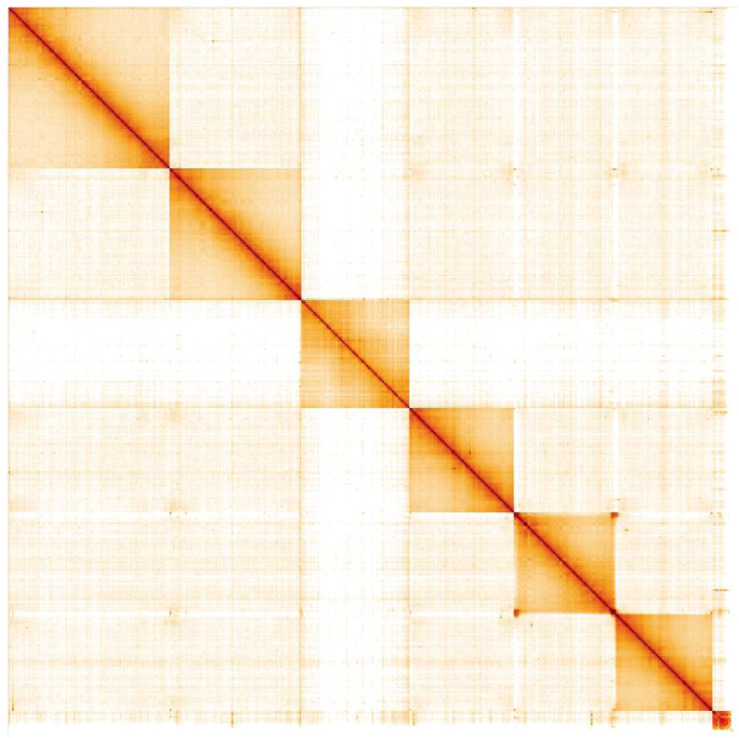
Genome assembly of *Acanthosoma haemorrhoidale*, ihAcaHaem1.1: Hi-C contact map. Hi-C contact map of the ihAcaHaem1.1 assembly, visualised in HiGlass. Chromosomes are arranged in size order from left to right and top to bottom. The interactive Hi-C map can be viewed at https://genome-note-higlass.tol.sanger.ac.uk/l/?d=SITVSHcSTwOqolCwz4HaZA.

The assembly has a BUSCO v5.2.2 ( Manni *et al.*, 2021) completeness of 99.2% (single 97.4%, duplicated 1.8%) using the hemiptera_odb10 reference set (n=954). While not fully phased, the assembly deposited is of one haplotype. Contigs corresponding to the second haplotype have also been deposited.

**Table 2.** Chromosomal pseudomolecules in the genome assembly of *Acanthosoma haemorrhoidale, ihAcaHaem1.1*.

INSDC accession	Chromosome	Size (Mb)	GC%
OV884011.1	1	193.55	35.5
OV884012.1	2	157.4	35.3
OV884013.1	3	125.66	35.4
OV884014.1	4	120.69	35.3
OV884015.1	5	116.64	35.2
OV884009.1	X	129.25	35.7
OV884010.1	Y	4.79	36.8
OV884016.1	MT	0.02	24.2
-	Unplaced	17.64	37.8

## Methods

### Sample acquisition and nucleic acid extraction

A single *A. haemorrhoidale* specimen (ihAcaHaem1) was collected using a pot from Wytham wood, Berkshire, UK (latitude 51.772, longitude -1.336) by Liam Crowley (University of Oxford). The specimen was identified by Liam Crowley and snap-frozen on dry ice.

DNA was extracted at the Tree of Life laboratory, Wellcome Sanger Institute. The ihAcaHaem1 sample was weighed and dissected on dry ice with tissue set aside for Hi-C sequencing. Abdomen tissue was cryogenically disrupted to a fine powder using a Covaris cryoPREP Automated Dry Pulveriser, receiving multiple impacts. Fragment size analysis of 0.01–0.5 ng of DNA was then performed using an Agilent FemtoPulse. High molecular weight (HMW) DNA was extracted using the Qiagen MagAttract HMW DNA extraction kit. Low molecular weight DNA was removed from a 200-ng aliquot of extracted DNA using 0.8X AMpure XP purification kit prior to 10X Chromium sequencing; a minimum of 50 ng DNA was submitted for 10X sequencing. HMW DNA was sheared into an average fragment size between 12–20 kb in a Megaruptor 3 system with speed setting 30. Sheared DNA was purified by solid-phase reversible immobilisation using AMPure PB beads with a 1.8X ratio of beads to sample to remove the shorter fragments and concentrate the DNA sample. The concentration of the sheared and purified DNA was assessed using a Nanodrop spectrophotometer and Qubit Fluorometer and Qubit dsDNA High Sensitivity Assay kit. Fragment size distribution was evaluated by running the sample on the FemtoPulse system.

### Sequencing

Pacific Biosciences HiFi circular consensus and 10X Genomics Chromium read cloud sequencing libraries were constructed according to the manufacturers’ instructions. Sequencing was performed by the Scientific Operations core at the Wellcome Sanger Institute on Pacific Biosciences SEQUEL II (HiFi) and Illumina NovaSeq 6000 (10X) instruments. Hi-C data were generated in the Tree of Life laboratory from head/thorax tissue of ihAcaHaem1 using the Arima v2 kit and sequenced on a NovaSeq 6000 instrument.

### Genome assembly

Assembly was carried out with Hifiasm ( Cheng *et al.*, 2021); haplotypic duplication was identified and removed with purge_dups ( Guan *et al.*, 2020). One round of polishing was performed by aligning 10X Genomics read data to the assembly with longranger align, calling variants with freebayes ( Garrison & Marth, 2012). The assembly was then scaffolded with Hi-C data ( Rao *et al.*, 2014) using SALSA2 ( Ghurye *et al.*, 2019). The assembly was checked for contamination and corrected using the gEVAL system ( Chow *et al.*, 2016) as described previously ( Howe *et al.*, 2021). Manual curation ( Howe *et al.*, 2021) was performed using gEVAL, HiGlass ( Kerpedjiev *et al.*, 2018) and Pretext. The mitochondrial genome was assembled using MitoHiFi ( Uliano-Silva *et al.*, 2021), which performs annotation using MitoFinder ( Allio *et al.*, 2020). The genome was analysed and BUSCO scores generated within the BlobToolKit environment ( Challis *et al.*, 2020). Table 3 contains a list of all software tool versions used, where appropriate.

**Table 3.** Software tools used.

Software tool	Version	Source
Hifiasm	0.15.3-r339	Cheng *et al.*, 2021
purge_dups	1.2.3	Guan *et al.*, 2020
SALSA2	2.2	Ghurye *et al.*, 2019
longranger align	2.2.2	https://support.10xgenomics.com/genome-exome/ software/pipelines/latest/advanced/other-pipelines
freebayes	1.3.1-17- gaa2ace8	Garrison & Marth, 2012
MitoHiFi	2.0	Uliano-Silva *et al.*, 2021
HiGlass	1.11.6	Kerpedjiev *et al.*, 2018
PretextView	0.2.x	https://github.com/wtsi-hpag/PretextView
BlobToolKit	3.0.5	Challis *et al.*, 2020

### Ethics/compliance issues

The materials that have contributed to this genome note have been supplied by a Darwin Tree of Life Partner. The submission of materials by a Darwin Tree of Life Partner is subject to the Darwin Tree of Life Project Sampling Code of Practice. By agreeing with and signing up to the Sampling Code of Practice, the Darwin Tree of Life Partner agrees they will meet the legal and ethical requirements and standards set out within this document in respect of all samples acquired for, and supplied to, the Darwin Tree of Life Project. Each transfer of samples is further undertaken according to a Research Collaboration Agreement or Material Transfer Agreement entered into by the Darwin Tree of Life Partner, Genome Research Limited (operating as the Wellcome Sanger Institute), and in some circumstances other Darwin Tree of Life collaborators.

## Data availability

European Nucleotide Archive: Acanthosoma haemorrhoidale (hawthorn shieldbug). Accession number PRJEB47321; https://identifiers.org/ena.embl/PRJEB47321.

The genome sequence is released openly for reuse. The *A. haemorrhoidale* genome sequencing initiative is part of the Darwin Tree of Life (DToL) project. All raw sequence data and the assembly have been deposited in INSDC databases. The genome will be annotated and presented through the Ensembl pipeline at the European Bioinformatics Institute. Raw data and assembly accession identifiers are reported in Table 1.

## Author information

Members of the University of Oxford and Wytham Woods Genome Acquisition Lab are listed here : https://doi.org/10.5281/zenodo.6418202.

Members of the Darwin Tree of Life Barcoding collective are listed here: https://doi.org/10.5281/zenodo.6418156.

Members of the Wellcome Sanger Institute Tree of Life programme are listed here: https://doi.org/10.5281/zenodo.6418327 .

Members of Wellcome Sanger Institute Scientific Operations: DNA Pipelines collective are listed here: https://doi.org/10.5281/zenodo.5746904.

Members of the Tree of Life Core Informatics collective are listed here: https://doi.org/10.5281/zenodo.6125046.

Members of the Darwin Tree of Life Consortium are listed here: https://doi.org/10.5281/zenodo.6418363.
