# Supplementary material for: The genome sequence of the hawthorn shieldbug, Acanthosoma haemorrhoidale (Linnaeus, 1758)
Source: Wellcome Open Res. 2022 Jul 5;7:178. [Version 1] doi: 10.12688/wellcomeopenres.17926.1 (PMC9971695; doi:10.12688/wellcomeopenres.17926.1)
Supplement: Peer review history

# Peer review: Reviewer response for version 1

**DOI:** 10.21956/wellcomeopenres.19864.r53971

**Published:** 2023-02-10

**Authors:** Vani Brahmachari, Surbhi Kohli

**Affiliations:** 1 University of Delhi, Delhi, India; 2 Department of Biochemical Engineering and Biotechnology, IIT (Indian Institute of Technology), Delhi, India

The authors provide a comprehensive account of the genome assembly of the hawthorn shield bug. The authors highlight the pheromone production by males and its reception by females as well as the host-symbiont relationship in hawthorn shield bugs as interesting features and hence a motivation for elucidating the genome sequence. However, there are no further comments on these aspects. Though it may be reserved for a detailed study, a comment on the indication of metabolic cooperation, if any, would be interesting. 

 A combination of PacBio sequencing and HiC has led to chromosomal level assembly of several insect genomes and has been effectively utilized in this manuscript.

 The methods are clearly described and the outcomes are provided in the figures. I appreciate the interactive figures, however I could not access interactive Hi-C map, “ **No such uuid” **comes up at the site.

 Minor comments:

1. The N50 value mentioned of scaffolds mentioned on page 3 is different from that in Table 1.
2. In Fig. 3, three categories of scaffolds are given (Total, No hit and Arthropod). Were the symbiont sequences and horizontal transfer gene scaffolds removed from the assembly? Please clarify.
3. As mentioned in the manuscript the authors were able to generate two different haplotypes of the assembly; which is the tool used for resolving the haplotype?
4. Following clarification on the above comments the manuscript is approved. 

Are sufficient details of methods and materials provided to allow replication by others?

Partly

Is the rationale for creating the dataset(s) clearly described?

Yes

Are the datasets clearly presented in a useable and accessible format?

Yes

Are the protocols appropriate and is the work technically sound?

Yes

Reviewer Expertise:

Epigenetics, mining epigenetic regulatory factors from the genome.  Exploring genomic imprinting mechanisms using the coccid insect system, namely the mealybugs.

We confirm that we have read this submission and believe that we have an appropriate level of expertise to confirm that it is of an acceptable scientific standard.

# Peer review: Reviewer response for version 1

**DOI:** 10.21956/wellcomeopenres.19864.r53978

**Published:** 2023-01-31

**Authors:** Kristen Panfilio

**Affiliations:** 1 University of Hohenheim, Stuttgart, Germany; 2 University of Warwick, Coventry, UK

As the Darwin Tree of Life (DToL) genome sequencing project continues to produce high-quality (chromosome-scale) genome assemblies for eukaryotic species of the British Isles, I welcome here the documentation of a genome assembly for the shieldbug *Acanthosoma haemorrhoidale*. The bugs (the Hemiptera, including aphids, cicadas, and the true bugs) are the most species-rich order of hemimetabolous insects. Increasing genomic resources for this major animal group will support ongoing research on biodiversity and many aspects of insect biology and ecological interactions, extending resources beyond the already well-sampled Holometabola and pest species of Hemiptera.

 I understand that DToL prioritizes rapid dissemination of genome data according to a standard template, and the Wellcome Open Research format of the Data Note is a great fit for this. However, having been asked to review this specific contribution, I find that there are some aspects of presentation for dataset reproducibility, rigor, and readability that should be improved.

**Concern leading to status of “approval with reservations”:**

 Documentation of species identification is insufficient. In the Data Note, it is based solely on a low-magnification image of the sequenced individual (Figure 1) and a methods statement that the specimen was collected and identified “by Liam Crowley (University of Oxford)”. This is insufficient, as there is no mention of a type specimen accession, barcoding identification, or a cited reference for taxonomic expertise. At present, identification of wild-caught individuals for future research is not reproducible, unless Liam Crowley is a resource available on demand to the scientific community! Also, please ensure that the Figure 1 image is of the highest quality, provide a scale bar (should be possible, given the inclusion of a standard collection tube in the image), and crop unneeded white space to maximize size of the insect within the image.

**Readability issues, including technical documentation, general clarity, and completeness (rigor) of scientific presentation:**

 The final paragraph of the Background states that the karyotype consists of ten autosomes and the X and Y sex chromosomes, and this is supported by identification of seven unique chromosomes in the current assembly (Figure 5), but it would be easier to reconcile these facts if the Background comment were reworded slightly (suggestion in all caps) to “the diploid (2N) karyotype of *A. haemorrhoidale* to be 12, comprising FIVE AUTOSOMAL PAIRS and two sex chromosomes…”. Also, include the year immediately after the author names for clear attribution at the beginning of this sentence, particularly since multiple references are cited together at the end.

 In the “Sequencing” section, please specify reagent (chemistry) versions for the HiFi, 10X, and Hi-C work, similar to the software details provided in Table 3. Who is the manufacturer of the Arima kit?

 The presentation of Figures 2-5 is inadequate for a non-bioinformatic audience, such as entomologists and molecular geneticists. In-text mention is confined to a single stub, batch parenthetical citation early in the “Genome sequence report” section. The figures and their legends are devoid of biological information and apparently only present pipeline-generated statistical visualization features with no wider context or attempt to customize appearance in a fashion appropriate to this species’ assembly values. A few sentences in the main text should make clear what each figure presents. Even if the figures themselves may have interactive online versions, they should be annotated and intelligible in the Data Note itself, with consistent and legibly sized in-figure text legends and text labels that are not merely pipeline designations (e.g., “CAKNEZ01” in Figures 3 and 4).

 In Figure 2, there were many elements that I could not interpret. The lower left “Scale” is of unclear value. The upper right BUSCO pie chart is too small and disproportionately tiny compared to the main pie chart. Customarily, these elements should be distinguished as figure panels (a) and (b), not simply dumped in the same graphical space. For the BUSCO chart, there seems to be an inappropriate distinction between complete and duplicated, as the latter is a subset of the former in Table 1. Also, when multiple features of the BUSCO pie chart will be impossibly small to visually distinguish (2 features each <1%), what is the value of this chart in the first place, compared to the clear information already provided in Table 1 and with an interactive hyperlink in the Table 1 footnote? For the main chart, the multiple instances of red and grey referred to in the figure legend were ambiguous, and I could not reconcile these elements with the actual image appearance: please provide in-figure text labels. For example, I cannot tell which is the “dark grey” for chromosome lengths and which is the “pale grey spiral” for cumulative chromosome counts. The outer blue tracks to indicate GC content may be a standard pipeline output, but it is of no clear visual value here. Rather, the Figure 2 in-figure legend for this feature should be explicitly given for GC content statistics in Table 1. Elsewhere, there appears to be a typo, as the main text and Table 1 cite 72 scaffolds in the assembly, whereas the upper left legend for Figure 2 lists 73. If there are only 7 chromosomes, why is “chromosome count” given on a log scale? In general, the main pie chart left me none the wiser about the assembly, where I only gleaned key information from the in-figure legends with numerical values or, partially, from the main figure caption.

 Compared to a simple report that GC content is 35.5%, what is the value of Figure 3? In its present state, it appears to be an unaltered pipeline output, but unhelpful for the uninitiated (see comments above).

 Please amend Figure 4 so that the plot itself and in-figure text are appropriately sized. At present the chart is unnecessarily large for its complexity and content, while the in-figure text is too small for the axes and legend (and see above on using appropriate legend text labels). To make this chart useful, in the legend or main text please report what percent of the total assembly can be designated as arthropod-specific, attributable to another phylum (does the pipeline support identification of microbial content? – see below), or with no assigned phylum. Also, as BUSCO focuses on protein coding genes and this assembly has not yet been formally annotated with an official gene set, please comment on what fraction of the assembly is assessed by this method (the technical reference to “buscogenes taxrule” is not informative for me).

 I value Figure 5, but there is a complete absence of axis labels or heatmap color code legend for these Hi-C data. It would also be helpful if in-figure annotations indicated the X and Y chromosomes. Alternatively, if this is correct, state in the figure legend that “linkage groups corresponding to chromosomes are presented from top left to lower right in order of descending size, from Chromosome 1 to Y, as listed in Table 2, with the mitochondrial assembly not shown”. Also, the link to the interactive version of this figure seems to be broken (“No such uuid”) – please amend. 

 For the “Genome assembly” section, the main text link to “Pretext” on GitHub requires more detailed documentation and citation to indicate which exact methods were used (which versions) in a Data Note generated at a specific point in time and that has a permanent DOI. For the main text citation of Table 3, what does “where appropriate” include or exclude (or, what is the purpose of this caveat)?

 Please report the actual assembly metrics associated with the analyses for contamination and mitochondrial genome assembly. As noted by Andrew J. Mongue in his peer review report (5 August 2022), it is a notable omission, after introductory information on interest in microbiomes, that there is no main text reporting on these findings. I was interested to note that the abdomen – the predominant location of microbiome components associated with reproductive and digestive anatomy – was sequenced by HiFi but excluded for Hi-C, which presumably provides a basis for at least an initial explicit statement on which fraction of the unplaced assembly (17.6 Mb) may be due to specimen heterozygosity, microbial content, or other sources.

**Taxonomic corrections:**

 In the article Background, first paragraph, please correct the spelling of the superfamily name “Pentatomoid” (the “-to-“ is missing).

 The peer review report of this article by Andrew J. Mongue refers to aphids and mealybugs as “true bugs”, but this is incorrect. The term “true bugs” applies to the monophyletic Heteroptera, which does include the species presented here ( *Acanthosoma haemorrhoidale*), while aphids and mealybugs belong to the distinct lineage Sternorrhyncha, sometimes (formerly) regarded as a part of the paraphyletic Homoptera (see, for example, Figure 2 in: Panfilio *et al.*, 2018 ^1^).

Are sufficient details of methods and materials provided to allow replication by others?

Partly

Is the rationale for creating the dataset(s) clearly described?

Yes

Are the datasets clearly presented in a useable and accessible format?

Partly

Are the protocols appropriate and is the work technically sound?

Yes

Reviewer Expertise:

insects; Hemiptera; Coleoptera; comparative genomics; evolution of development; molecular evolution; macroevolutionary processes; developmental genetics; molecular genetics; gene regulatory networks; transcription factors; transgenesis; cell and tissue development; fluorescence microscopy; live cell imaging

I confirm that I have read this submission and believe that I have an appropriate level of expertise to confirm that it is of an acceptable scientific standard, however I have significant reservations, as outlined above.

## References

[1] : By land, air, and sea: hemipteran diversity through the genomic lens. *Curr Opin Insect Sci* .2018;25: 10.1016/j.cois.2017.12.005 106-115 10.1016/j.cois.2017.12.005 29602356

# Peer review: Reviewer response for version 1

**DOI:** 10.21956/wellcomeopenres.19864.r51880

**Published:** 2022-08-05

**Authors:** Andrew J. Mongue

**Affiliations:** 1 Institute of Evolutionary Biology, The University of Edinburgh, Edinburgh, UK

The authors report the genome assembly of the hawthorn shield bug with brief background and detailed methodology of sequencing and assembly. I understand this reporting format is designed to be concise yet informative and I commend the authors on the level of rigor in describing the process from DNA extraction through to finished assembly. As this and other recent genome assembly papers have demonstrated, the combination of PacBio HiFi and HiC sequencing is an almost guaranteed success for generating highly contiguous insect genomes. Given that this approach seems to be so powerful, it is all the more important that researchers document the methodology in detail so that others can replicate this success. I appreciated the table listing tools and their versions as an example of how to effectively highlight methodological details. Likewise, the accession numbers appropriately link to data that are now available, so these resources have immediate value to the community as well.

 I have only two comments for clarification and these focus on the species background context.

 Firstly, the authors contrast shield bug pheromone production to that of Lepidoptera, stating that female Lepidoptera produce pheromones to attract males. While this is true of some groups within the order, many species behave much like the bugs described here: males produce pheromones to entice females (see milkweed butterflies as a specific example and generally species in which males have a hair-pencil organ). Please clarify the text on this point.

 Secondly, the authors mention the potential for sequencing data to reveal symbiont relationships that the hawthorn shield bug has with bacteria. Host-microbe interactions are particularly well-studied in true bugs like aphids and mealybugs, so I can see the value in this new datapoint for comparison. As such, I was surprised that symbiont screening is never directly mentioned again in the methods or results. Perhaps this is a structural choice from the Tree of Life initiative and these data will be reported elsewhere. Given the interest in hemipteran symbionts from the research community, however, I feel it would be good to include at least a follow-up sentence directing the interested reader to the appropriate resources.

 I also noticed two small grammatical errors to correct:

- “The hawthorn shield bug…is a large Pentamoid shield bug, easily recognisable by their size” should be “easily recognisable by its size”.
- “The species is common on hawthorn…but are also found in mixed woodland” should be “but is also found in mixed woodland”.

Are sufficient details of methods and materials provided to allow replication by others?

Yes

Is the rationale for creating the dataset(s) clearly described?

Yes

Are the datasets clearly presented in a useable and accessible format?

Yes

Are the protocols appropriate and is the work technically sound?

Yes

Reviewer Expertise:

Insect genomics, population genetics, Lepidopteran and Hemipteran biology

I confirm that I have read this submission and believe that I have an appropriate level of expertise to confirm that it is of an acceptable scientific standard.
